# Multispectral autofluorescence characteristics of reproductive aging in old and young mouse oocytes

**DOI:** 10.1007/s10522-022-09957-y

**Published:** 2022-02-24

**Authors:** Jared M. Campbell, Saabah B. Mahbub, Michael J. Bertoldo, Abbas Habibalahi, Dale M. Goss, William L. Ledger, Robert B. Gilchrist, Lindsay E. Wu, Ewa M. Goldys

**Affiliations:** 1 ARC Centre of Excellence Centre for Nanoscale Biophotonics, Graduate School of Biomedical Engineering, University of New South Wales Sydney, Kensington, Sydney, NSW 2052 Australia; 2 Discipline of Women’s Health, School of Clinical Medicine, University of New South Wales Sydney, Sydney, Australia; 3 School of Medical Sciences, University of New South Wales Sydney, Sydney, Australia

**Keywords:** Multispectral, Hyperspectral, Autofluorescence, Oocyte, Reproductive healthcare, Ageing

## Abstract

Increasing age has a major detrimental impact on female fertility, which, with an ageing population, has major sociological implications. This impact is primarily mediated through deteriorating quality of the oocyte. Deteriorating oocyte quality with biological age is the greatest rate-limiting factor to female fertility. Here we have used label-free, non-invasive multi-spectral imaging to identify unique autofluorescence profiles of oocytes from young and aged animals. Discriminant analysis demonstrated that young oocytes have a distinct autofluorescent profile which accurately distinguishes them from aged oocytes. We recently showed that treatment with the nicotinamide adenine dinucleotide (NAD+) precursor nicotinamide mononucleotide (NMN) restored oocyte quality and fertility in aged animals, and when our analysis was applied to oocytes from aged animals treated with NMN, 85% of these oocytes were classified as having the autofluorescent signature of young animals. Spectral unmixing using the Robust Dependent Component Analysis (RoDECA) algorithm demonstrated that NMN treatment altered the metabolic profile of oocytes, increasing free NAD(P)H, protein bound NAD(P)H, redox ratio and the ratio of bound to free NAD(P)H. The frequency of oocytes with simultaneously high NAD(P)H and flavin content was also significantly increased in mice treated with NMN. Young and Aged + NMN oocytes had a smoother spectral distribution, with the distribution of NAD(P)H in young oocytes specifically differing from that of aged oocytes. Identifying the multispectral profile of oocyte autofluorescence during aging could have utility as a non-invasive and sensitive measure of oocyte quality.

## Introduction

The international trend across developed nations for delayed conception (Adamson et al. 2018) is increasingly conflicting with age-related barriers to female fertility (De Vos et al. 2014; Sauer 2015). Although assisted reproduction technologies (ART) such as in vitro fertilisation (IVF) can improve reproductive success, they are invasive and carry health risks (Kumar et al. 2011). Furthermore, the decline in oocyte integrity from the mid-thirties impacts the capacity for embryo development and viable pregnancies (De Vos et al. 2014; Sauer 2015). As such, by the time a woman reaches her early forties her chance of livebirth per IVF cycle is < 10% (Newman et al. 2020).

This creates a clear clinical need for an improved understanding of oocyte ageing and rejuvenation strategies, including technologies for longitudinal, non-invasive assessment. In current clinical practice, oocyte quality is determined by a trained assessor and chiefly looks for the presence of a clear, moderately granular cytoplasm, the absence of inclusions, a small perivitelline space with an unfragmented first polar body and a round clear zona pellucida (Setti et al. 2011). Despite this, a systematic review of the literature has shown that none of these features have unanimous prognostic value for predicating further developmental competence of oocytes (Rienzi et al. 2011). When complex systems with multiple features are applied, first polar body size, perivitelline space, refractile bodies (Setti et al. 2011) and the presence of a meiotic spindle can predict the odds of fertilization by intracytoplasmic sperm injection (ICSI), but does not accurately predict the primary outcomes of embryo implantation and birth (Petersen et al. 2009). Overall, the literature points to a mixed success for morphological assessment of oocytes for predicting fertilization and pregnancy, with the weight of evidence suggesting a minor impact (Ashrafi et al. 2015). As well as the implications for clinical decisions in the IVF setting, the inability to non-invasively predict oocyte competence is an impediment to research on the mechanisms of reproductive decline.

Impaired oocyte viability with increasing age is the greatest rate-limiting factor to female fertility, raising the need for therapeutic interventions that maintain and/or rejuvenate oocyte function. Key mechanisms for oocyte failure include genome instability, elevated reactive oxygen species (ROS), disrupted mitochondrial activity, and impaired fidelity during meiotic chromosome segregation (Greaney et al. 2017). These molecular defects are similar to the transformations which characterise somatic cells ageing, which also experience declining mitochondrial bioenergetics (Gomes et al. 2013; Campbell et al. 2020), genomic instability (Vijg and Suh 2013), and impaired chromosome segregation, ultimately resulting in growth arrest and senescence (Baker et al. 2004). One mechanism for age-related dysfunction is the depletion of nicotinamide adenine dinucleotide (NAD^+^) (Massudi et al. 2012), a key redox cofactor and enzyme substrate required for energy metabolism, DNA repair and epigenetic homeostasis. Addressing this decline by supplementation with NAD^+^ precursors has gained attention as a potential geroprotective strategy for preserving health into later life (Mills et al. 2016; Rajman et al. 2018). We and others recently showed that NAD^+^ levels are decreased in the oocytes of aged mice and that its repletion by supplementation with the metabolic precursor nicotinamide mononucleotide (NMN) rejuvenates aspects of reproductive function, including number of ovulated oocytes, meiotic competency, fertilisation and live births (Bertoldo et al. 2020; Miao et al. 2020). This suggests that restoration of late-life NAD+ levels could have the potential to restore aspects of female fertility during reproductive ageing.

Here, we apply a novel multispectral imaging technology to assess oocytes from our recent experiments on young mice, aged mice, and aged mice that have received NMN (Bertoldo et al. 2020). In this new analysis we further characterise the molecular impacts of ageing and NMN supplementation on oocyte biology and also pilot a novel, non-invasive tool for assessing oocyte quality. This is based on exploiting the autofluorescence of several intracellular metabolites, which can be excited to emit light at specific, sometimes uniquely characteristic, wavelength ranges (Campbell et al. 2019; Mahbub et al. 2019). Often regarded as interfering noise in immunofluorescent microscopy, this autofluorescence can directly reflect the internal biochemistry of an oocyte without the need for biopsy staining or fixation (non-invasive, non-harmful assessment being a crucial requirement for human reproductive therapy). The list of metabolites which exhibit autofluorescence includes key indicators of cellular redox state, such as the reduced form of nicotinamide adenine dinucleotide (NADH) and flavin adenine dinucleotide (FAD) (Gosnell et al. 2016a, b). These coenzymes serve as the principal electron donors and acceptors of oxidative phosphorylation. NADH and FAD are autofluorescent, with NADH having excitation maxima at 290 and 351 nm and emission maxima at 440 and 460 nm, while FAD has an excitation maximum at 450 nm with its emission maxima at 535 nm. Other related fluorophores, such as. NADPH and other members of the flavin family, have similar excitation/emission profiles and are therefore not spectrally distinct from NADH and FAD. As a result, these are collectively referred to in the field as NAD(P)H and flavins. By using a number of excitation wavelengths and assessing the resulting emissions across a similarly broad range we are able to build a multiplex data cube which can predict relative molecular composition (abundances) with far more precision than techniques which target the key wavelengths of these individual autofluorophores (Gosnell et al. 2016a, b; Mahbub 2017; Rehman et al. 2017, Mahbub et al. 2019). Imaging at multiple wavelengths can improve adjustments for ‘background’ fluorescence from other autofluorophores (Duong and Han 2013; Yang et al. 2014). In the past, we have successfully used this approach to non-invasively measure the abundance of key cellular fluorophores in various biological settings (Campbell et al. 2019; Habibalahi et al. 2019a, 2020; Mahbub et al. 2019) including in cell ageing (Campbell et al. 2020). Importantly, multispectral microscopy has been successfully used to discriminate between bovine embryos grown in low oxygen and high oxygen environments (Sutton-McDowall et al. 2017), the latter condition resulting in perturbed development. The use of a broad panel of excitation/emission wavelengths means that spectral signals beyond NAD(P)H and flavins can be collected, interrogated and matched to the profiles of known autofluorophores. As such we have additionally investigated age related changes in oocytes for; free NAD(P)H, protein bound NAD(P)H, flavins, and collagen.

## Methods

### Animals

Aged (12-months) and Young (4- to 5-weeks) C57BL/6 J females were maintained in groups of 5, in individually ventilated cages at 22 °C at 80% humidity, with *ad libitum* access to food and water. Water was acidified to pH 3 with HCl to minimize microbial growth. A 12 h light/dark cycle was maintained with lights on at 0700 and off at 1900. Data was collected opportunistically during experiments reported in (Bertoldo et al. 2020) which were approved by the UNSW Animal Care and Ethics Committee (ACEC), number 18/133A, which operates under the animal ethics guidelines of the National Health and Medical Research Council (NHMRC) of Australia.

NMN (GeneHarbor Biotechnologies) was given to a cohort of aged females in their drinking water (2 g/l) for 4 weeks. Both aged and young females were then treated with an intraperitoneal (i.p.) injection of pregnant mare serum gonadotrophin (PMSG; Folligon, Intervet, Boxmeer, Holland) to stimulate follicle growth followed by an i.p. injection of human chorionic gonadotrophin (hCG; Chorulon, MSD Animal Health, Australia) 46 h later to induce ovulation. Young females received 5 IU, while aged females received 10 IU of each gonadotrophin. Cumulus-oocyte complexes were collected from oviductal ampullae into HEPES-buffered α-minimum essential medium (α-MEM; GIBCO Life Technologies, Grand Island, NY) supplemented with 3 mg/ml bovine serum albumin (BSA; Sigma Aldrich. St Louis, MO), using a 27-guage needle 14–16 h after hCG injection in order to recover mature metaphase II (MII) oocytes. At least three mice were used per group. These were then stripped of their cumulus cells with hyaluronidase prior to imaging. Oocyte numbers were 27, 21 and 26 for Young, Old and NMN groups, respectively.

### Spectral microscopy

Oocytes were placed into equilibrated Hank’s balanced salt solution (HBSS) under paraffin oil in coverslip-bottomed dishes and maintained at 36.5 °C during imaging. Spectral microscopy was carried out with an adapted fluorescence IX81 Olympus microscope with a 40× oil objective (NA 1.15) a Prime95B™ sCMOS (Photometrics) camera operated below − 30 °C to reduce noise. Custom-designed LED illumination and light filtering enabled autofluorescence imaging at excitation/emission wavelength combinations (in nm) 345/414, 345/451, 345/575, 345/594, 490/594, 505/594, 358/414, 371/414, 377/ 414, 381/414, 358/451, 371/451, 377/451, 381/451, 358/575, 371/575, 377/575, 391/575, 406/575, 418/575, 437/575, 457/575, 469/575, 476/575, 490/575, 505/575; where excitation values are ± 5 nm and emissions are ± 20 nm.

### Autofluorophore unmixing

Linear mixed modelling (LMM) was used in this work to compare the measured spectral characteristics of the oocytes to reference spectra of specific fluorophores to find their abundance. In LMM the autofluorescent signals at each pixel are assumed to be a linear combination of a small number of endmember component spectra with weights corresponding to the concentration of the fluorophores responsible for these. Spectral channels for imaging were chosen to minimise spectral overlap between autofluorophores. We used an unsupervised algorithm Robust Dependent Component Analysis (RoDECA) to identify dominant endogenous fluorophores and their abundance. RoDECA has been shown to discriminate individual fluorophores, despite overlapping spectra, in the presence of image noise (Mahbub 2017; Mahbub et al. 2017b, 2019). For validation, the spectral characteristics of the extracted fluorophores in cells were compared to the known characteristics of the same chemically pure fluorophores and their abundance was calculated (relative concentration) (Keshava et al. 2000; Keshava and Mustard 2002; Keshava 2003; Mahbub et al. 2017a). Close correlation between reference spectra and unmixed, RoDECa spectra has been reported (Campbell et al. 2021). Protein bound NAD(P)H was distinguished from free NAD(P)H using the reference spectra of L-Malate Dehydrogenase (Sigma Aldrich #10127248001) bound to NADH (Sigma Aldrich # 10107735001) in Mops (Sigma Aldrich # M1254) (Rehman et al. 2017). The spectral shift between NADH and protein bound NADH had been observed in the deep UV region, which is carefully covered during the multispectral acquisition (Lakowicz et al. 1992; Rehman et al. 2017).

### Multispectral discrimination modelling

Multispectral discrimination modelling was performed using a variety of quantitative cellular image features including mean channel intensity, channel intensity ratio (Gosnell et al. 2016), color distribution (El Aziz et al. 2017), and textural features and many others (Zhu et al. 2017). Definitions of the features examined have been discussed in past works (Gosnell et al. 2016b; El Aziz et al. 2017; Zhu et al. 2017). Candidate features (ANOVA p < 0.005) were projected onto an optimal two-dimensional space created by discriminative analysis. This space maximized between-group distance and minimized within-group variance while reducing the dimensions of the feature vectors to two canonical variables (defined as a linear combination of the selected features) Habibalahi et al. 2019a, b, Habibalahi et al. 2020). A classifier was applied based on a linear predictor function which incorporated a set of weights obtained from the training process (Vapnik 2013; Gosnell et al. 2018).

### Statistics

Analyses were carried out in Matlab (R2017b). Data was non-parametric so the Mann-Whitney U test was used. For comparison of frequencies the Chi-square test was applied except were n < 5, in which case Fischer’s exact test was used. Data are presented as median values with 95% confidence intervals. Alpha was p ≤ 0.05.

## Results

**Fig. 1:**
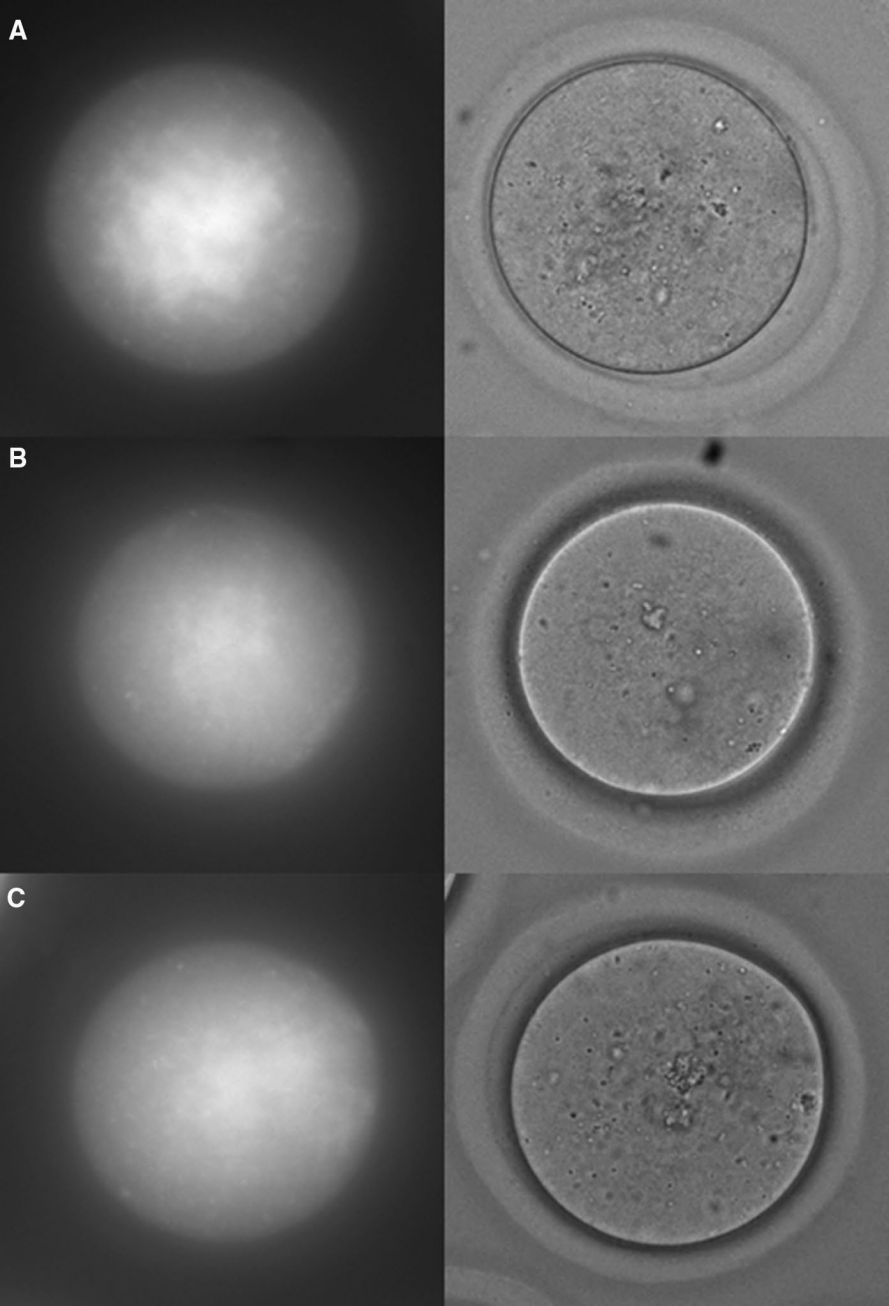
Spectral (channel 2) and brightfield representative images of oocytes from **a** young, **b** aged and **c** Aged mice treated with NMN

In a recent study of reproductive ageing, we collected hitherto unreported data on the spectral properties of oocytes (Bertoldo et al. 2020). We used 4–5 week old and 12-month old mice as a model for comparing young and reproductively aged oocytes, respectively. Mice were superovulated, oocytes collected and subject to multispectral autofluorescence imaging. A third group of aged animals was supplemented with the NAD+ precursor NMN, which we and others have shown improves oocyte function and reproductive outcomes (Bertoldo et al. 2020; Miao et al. 2020). Representative bright field images from these three groups of oocytes as well as corresponding spectral channel images are shown in Fig. 1A–C. Oocytes from the different age and treatment groups were not morphologically distinct.

### Spectral profile of oocyte age

**Fig. 2:**
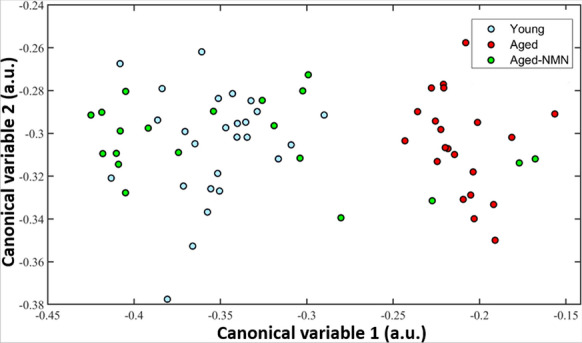
Modelling of multispectral feature data categorising oocytes into Young (blue), Aged (red) and Aged treated with NMN (green)

Multispectral discrimination modelling utilized quantitative cellular image features from the autofluorescent profiles of these oocytes (Fig. 1) to identify unique canonical variables that separated the signatures of young and aged oocytes (Fig. 2). Our classifier applied to this data achieved complete separation of young from aged oocytes (Fig. 2) however it should be noted that due to the small number of oocytes produced by aged mice our data set was limited and cross validation with a testing data set could not be undertaken. We then applied this model to oocytes from aged mice treated with NMN and found that the majority of these had multispectral feature data which resulted in their being classified with oocytes from young mice (Fig. 2A, 85%), with only a small subset being classified as having come from aged control mice (15%). This finding indicates that in most cases, NMN supplementation restored a youthful autofluorescent spectral profile to oocytes from aged animals, matching our previous findings of improved oocyte quality and reproductive function from this intervention (Bertoldo et al. 2020). These findings highlight the possibility of multispectral imaging as an indicator of oocyte quality.

### Fluorophore concentrations and distribution

**Fig. 3:**
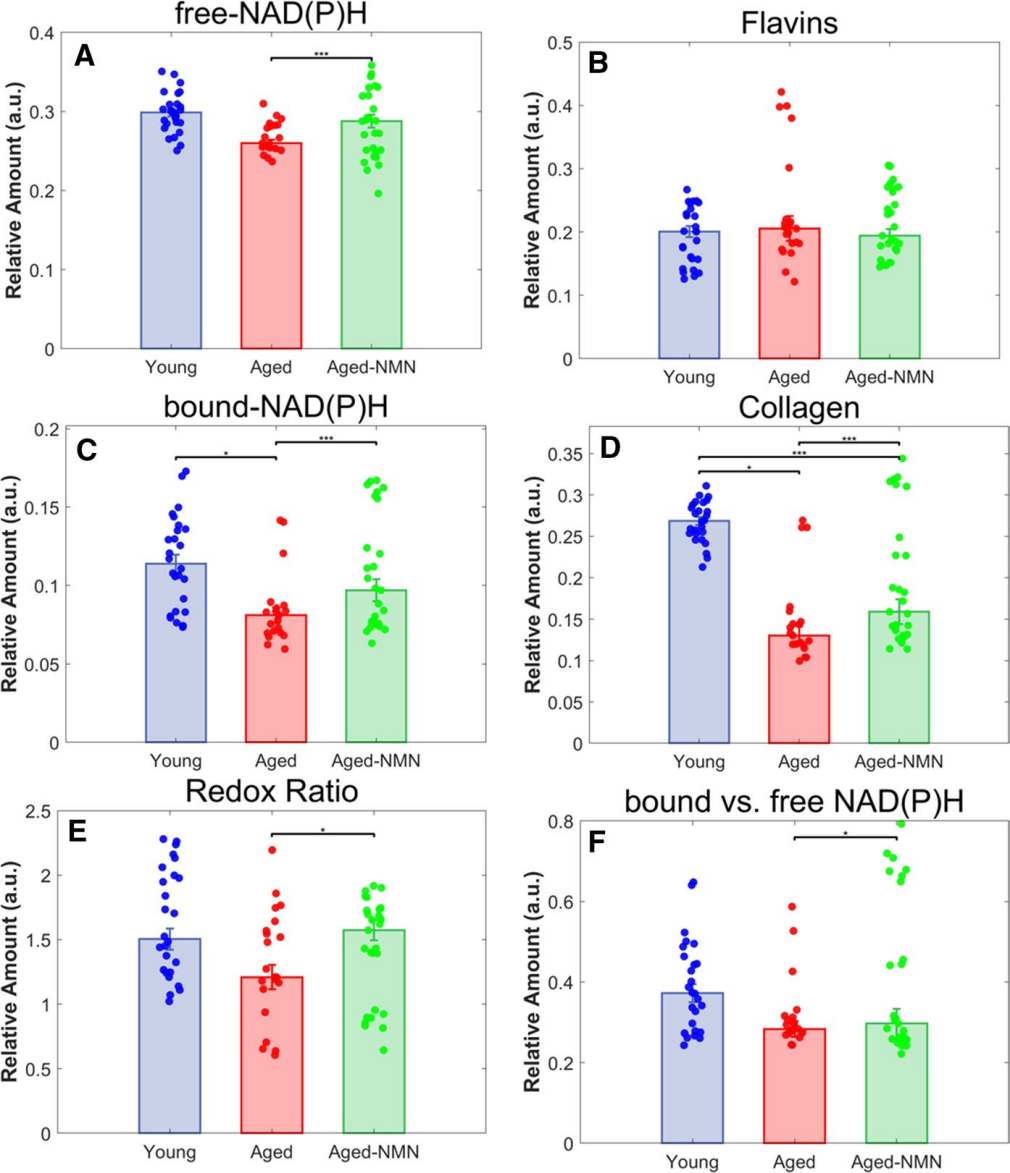
Relative abundance of **A** NAD(P)H, **B** Flavins, **C** protein bound NAD(P)H (b-NAD(P)H), **D** Collagen, **E** Redox ratio (NAD(P)H vs.Flavins) and **F** protein bound vs. free NAD(P)H. * indicates p < 0.05, *** indicates p < 0.0001

Spectral unmixing was applied to characterize the properties of individual oocytes by quantifying the relative concentrations of the most prevalent fluorophores giving rise to the general spectral profile of oocyte autofluorescence. We previously reported the total NAD(P)H levels for these oocytes in Bertoldo et al. 2020 (Bertoldo et al. 2020), here we report the outcomes for protein bound and free NAD(P)H (Fig. 3A). We found that the relative abundance of free NAD(P)H (Fig. 3A) and the corresponding redox ratio (free NAD(P)H/Flavins Fig. 3E)) were both significantly (p < 0.05) elevated in aged NMN treated animals compared to untreated aged controls. The spectral signatures for protein bound NAD(P)H and collagen were also identified, with bound NAD(P)H being significantly (p < 0.05) elevated in young compared to aged oocytes (Fig. 3C), and well NMN treated aged mice compared to untreated aged mice (p < 0.0001; Fig. 3C). When the ratio of bound to free NAD(P)H was examined, the difference was only significant (p < 0.05) for NMN treated aged mice compared to untreated aged (Fig. 3F). Interestingly, collagen content was elevated in the young (p < 0.0001) and NMN treated aged groups (p < 0.05) compared to the untreated aged groups (Fig. 3D).

**Fig. 4:**
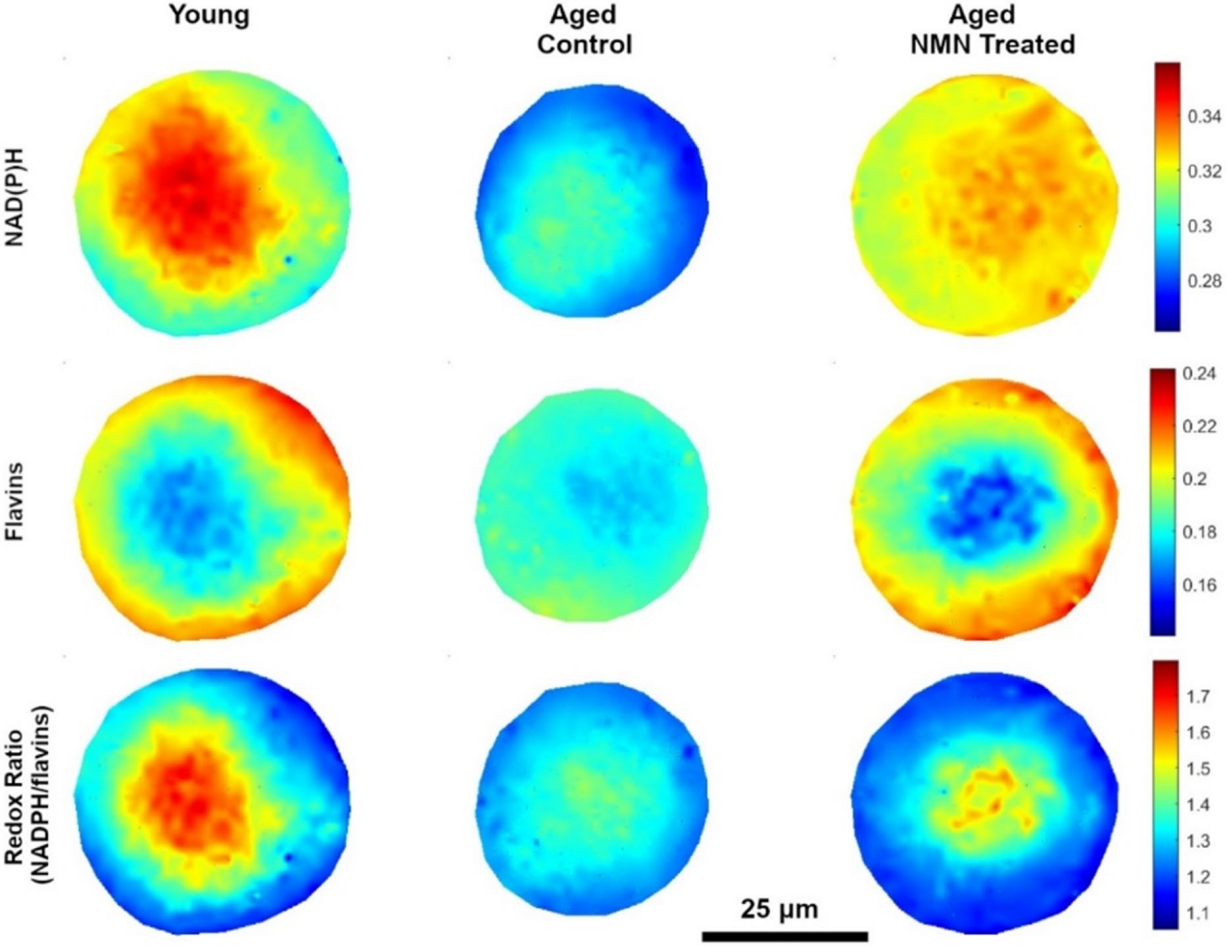
Heatmap for NAD(P)H, flavins, and Redox ratio for Young, Aged control, and Aged + NMN treatments

We were also able to reconstruct cellular maps of key fluorophores (Fig. 4) and observed that spatial differences in the distribution of areas of low and high abundance of fluorophores within oocytes may differ between oocyte groups. Such an occurrence would not necessarily be evident in the comparison of mean abundances. As such, this observation was investigated quantitatively by mapping the abundance spectra from the middle of a central region of distinct fluorophore abundance visible across all maps (Fig. 4) to the oocyte boundary. A linear vector was fitted for each spectrum to avoid nonlinear distribution of the abundance due to sub-cellular spatial distribution and a gradient was then calculated with respect to the distance (µm).

$$m=\frac{difference\, of \,spectral \,intensity\, \left(normalized\, abundunce\right)}{difference\, of \,distance \left(\mu m\right)}$$

**Fig. 5:**
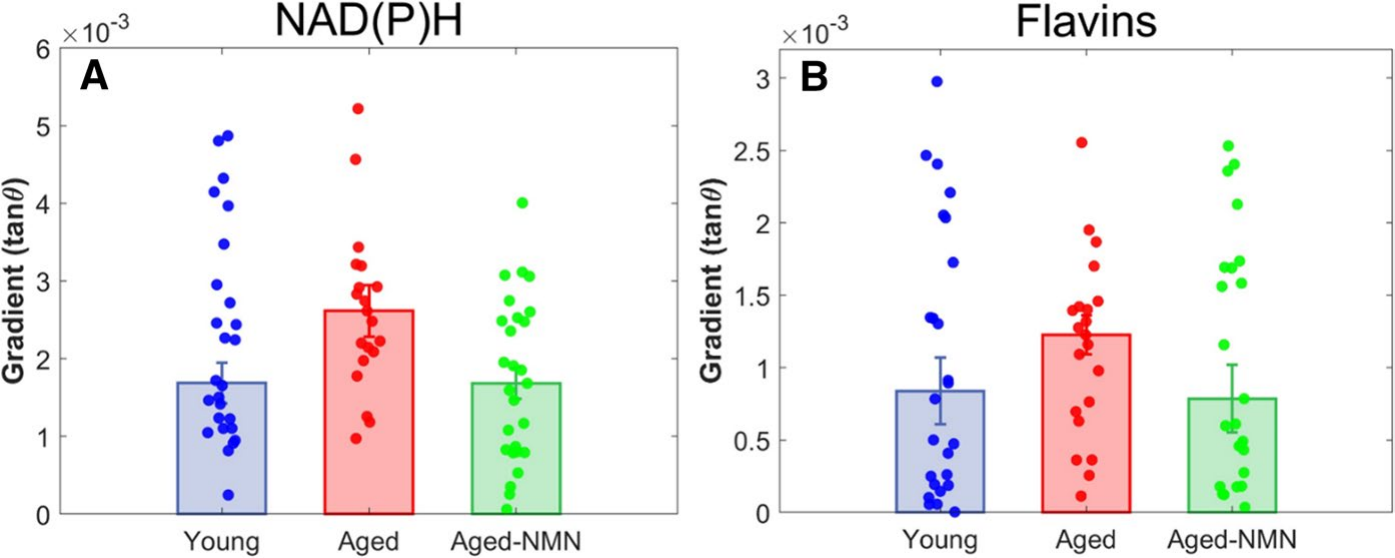
Spatial distribution of flavins and NAD(P)H assessed by concentration gradient of flavins (**a**) and NAD(P)H (**b**) within Young, Aged and Aged + NMN oocytes. ** indicates p < 0.005

As such, lower *m* values indicate a slow, gradual concentration increase, while larger *m* values indicate steeper gradients. A Mann Whitney U test was then performed for the *m* values, and we found that both young and aged + NMN groups had more gradual gradients for their overall fluorescence compared to oocytes from untreated, aged animals. This assessment was repeated for the distribution of free NAD(P)H and flavins specifically, and although no statistically significant differences were found between all groups for flavins, young oocytes had a significantly (p < 0.005) lower gradient of NAD(P)H compared to Aged oocytes (Fig. 5).

**Fig. 6:**
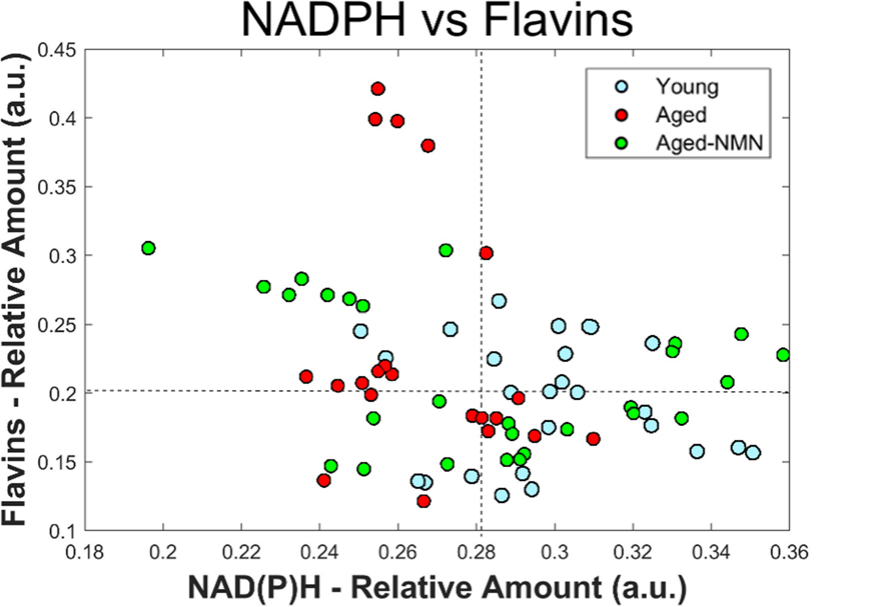
Free NAD(P)H plotted against flavin concentration for young, aged and aged treated with NMN oocytes

We next calculated the global mean for NAD(P)H and flavins within individual oocytes, and categorized them as high or low, based on their abundance relative to the global mean for all oocytes in their class (Table 1; Fig. 6). NMN treatment increased the proportion of oocytes in the high NAD(P)H category relative to both young (p = 0.05) and aged oocytes (p = 0.002), as determined by chi-square test. The difference between young and aged oocytes was not significant (Table 1). Further, while no difference was seen for flavins alone, a higher number of oocytes from NMN treated aged animals simultaneously had high NAD(P)H and flavins (p = 0.05 and p = 0.006 compared to young and aged, respectively Table 1).

**Table 1:** High and low NAD(P)H and flavins in individual oocytes

	Young	Aged	NMN
High NAD(P)H	14 (52%)	6 (29%)	20 (77%)
High Flavins	13 (48%)	11 (52%)	14 (54%)
High NAD(P)H + Flavins	5 (19%)	1 (5%)	11 (42%)
Sample (N)	27	21	26

## Discussion

In this study we found average differences in the abundance of distinct fluorophores between oocyte groups, with a large degree of variance in the characteristics of individual oocytes (Fig. 6). Our application of the general spectral signature built from the full multispectral imaging dataset was able to accurately discriminate oocytes from young animals and oocytes from reproductively aged animals (Fig. 2). The observation that oocytes from aged animals treated with NMN, which we previously showed improves aspects of fertility, were largely classified with oocytes from young animals is promising for this strategy, although conclusions are constrained by the lack of an appropriate testing dataset for validation. Oocytes with high free NAD(P)H and oocytes with both high NAD(P)H and flavins were most abundant from aged animals treated with NMN. Moreover, oocytes from young animals had a smoother overall spectral distribution than oocytes from aged, with NMN treatment restoring this characteristic. The distribution of free NAD(P)H was also shown to differ between oocytes from young and aged mice (Fig. 5), although the impact of NMN supplementation was not significant. Protein bound NAD(P)H and intracellular collagen have not previously been investigated in oocytes, and we show here that both were depressed in oocytes from aged mice compared to young. Interestingly, these were increased by NMN supplementation, suggesting that they may be useful biomarkers of quality. A decreased ratio of NAD(P)H to FAD has previously been observed in human oocytes that have undergone vitrification relative to fresh oocytes (Nohales-Corcoles et al. 2016), suggesting that this could act as a new marker of oocyte health. Other autofluorophores, such as lipofuscin (which accumulates in cells with age) and tryptophan (an NAD^+^ precursor) may have also contributed to the signal used to discriminate oocyte age, but their specific signature could not be unmixed by the present system.

With an increased age of reproduction resulting in increased use of IVF, there is a need to develop new methods to identify oocytes which are most suitable for IVF and implantation. These should ideally be non-invasive, however clinical practice relies on morphological assessment by a trained evaluator, which is highly subjective, poorly reproducible, and commonly groups oocytes with different developmental competencies in the same morphological categories (Petersen et al. 2009; Rienzi et al. 2011). Fluorescence lifetime imaging microscopy (FLIM), another fluorescence imaging technique for the assessment of autofluorescence, has previously been used to measure the metabolic co-enzymes NADH and FAD in oocytes from wildtype and knockout (*Clpp*) mice (Tejera et al. 2011). Unlike FLIM, our imaging technique was not able to discriminate between NADH and NAD(P)H, or isolate FAD from the greater flavin family. It was found that their NADH and FAD concentrations differed significantly between *Clpp* knockout and wildtype oocytes (Tejera et al. 2011). In another study, the application of FLIM to the assessment of NADH and FAD was able to discriminate oocytes whose metabolisms had been perturbed by the lactate dehydrogenase inhibitor oxamate or the mitochondrial transport chain Complex I inhibitor rotenone (Sanchez et al. 2019). Clinically sensitive assessment of oocyte quality within individuals is unlikely to be achieved solely through the quantification of just these two metabolic coenzymes—despite metabolism being a key determinant of embryo health (Thompson et al. 2016; Bertoldo et al. 2020).

An additional research priority for an increasing age at first pregnancy is to identify new interventions that rejuvenate oocyte quality, both for the purpose of increasing reproductive success and for ensuring the health of subsequent from dysfunction in both female (Cimadomo et al. 2018) and male (Campbell and McPherson 2019) gametes. In particular, mitochondria are maternally transmitted in mammals. As such, mitochondrial dysfunctions present in the oocyte can be directly passed down and cause congenital defects in offspring (Wu et al. 2015; Saben et al. 2016; Wei et al. 2019), including metabolic disease (Saben et al. 2016). We found that supplementation with the NADH precursor NMN significantly improved oocyte metabolic activity, with an increase in both free and protein bound NAD(P)H, as well as an increased redox ratio, relative to aged oocytes from mice that did not receive NMN (Fig. 3).

As mentioned previously, a key limitation of our study is that while a training dataset was used to develop criteria to separate young from aged oocytes, we are yet to validate this using an independent dataset. Additionally, the translatability of these findings is limited by the fact that they were made in mice. Although they have been found to be a good model for human reproduction under many circumstances it has been noted that full generalisation cannot be expected (Neuber and Powers 2000; Ménézo and Hérubel 2002), and results do, of course, need to be replicated in humans for implementation. Another important limitation is that while we are using maternal age as a classifier for presumed oocyte integrity, this classification may not correlate to true viability and reproductive potential. In future studies, where our classifiers would be validated on an independent dataset, the reproductive potential of each oocyte would be tested through IVF and transfer to young recipient females to test their reproductive success, enabling correlation of hyperspectral readings to oocyte function.

This direct evidence of sensitivity to viability will be needed for clinical translation of the technology where heterogeneity between oocytes is notably greater due to oocytes originating from follicles at varying stages of atresia and subordination, even in young women. Such translation would need to be undertaken in human oocytes in the context of IVF treatment, and with extreme caution due to the possibility of phototoxicity (Icha et al. 2017). Although we have shown that the exposure of embryos to our multispectral imaging system does not impact their developmental competence (Tan et al. 2021), this will need to be validated in mouse oocytes as a first step towards translation.Demonstrating that this technology has the potential to be applied to the non-invasive assessment of oocyte viability and quality requires a careful evaluation of phototoxicity—wherein high energy and, particularly, short, UV wavelength light causes the degradation of DNA and/or proteins (Icha et al. 2017). However, our recent work has shown, using embryo development and livebirth outcomes, that reproductive material is not overtly compromised by the wavelengths/intensities used by our system (Tan et al. 2021); furthermore many more dangerous UV wavelengths can be eliminated while retaining considerable capacity for biological characterization (Habibalahi et al. 2020). It should also be noted that male factor effects can have a major impact on embryo development and offspring health (Campbell et al. 2015; Campbell and McPherson 2019), with greater heterogeneity existing in the clinical scenario than among laboratory mice.

Efforts to develop more sensitive indicators of oocyte developmental potential have previously been limited by the strict necessity of absolute non-invasiveness. Although embryos can be biopsied for the detection of chromosomal abnormalities or genetic disorders, no such direct processes can be undertaken for oocytes which consist of a single cell. Alternative indirect methodologies for estimating oocyte quality have attempted to extrapolate from its microenvironment, including measuring oxygen consumption (Tejera et al. 2011), oocyte secreted factors (Gilchrist et al. 2008), and assessment of the cumulus cell transcriptome (Liu et al. 2018). If further work supports the potential of this technology, and indicates a lack of photo-toxicity, it may be translated to the sensitive assessment of oocyte viability, which would greatly improve human reproductive therapy and research into age-associated reproductive decline.

Multispectral assessment of oocyte autofluorescence has shown to be a sensitive marker of oocyte age. Together, future work testing the validity of this method on an independent dataset, including on human oocytes, and establishing a lack of photo-toxicity could be used as a new tool for investigating the biology of reproductive ageing.

## Data Availability

Data and code will be made available on reasonable request to the corresponding author.
